# Supplementary material for: Glypicans define unique roles for the Hedgehog co-receptors boi and ihog in cytoneme-mediated gradient formation
Source: eLife. 2021 Aug 6;10:e64581. doi: 10.7554/eLife.64581 (PMC8410076; doi:10.7554/eLife.64581)
Supplement: Source code 1. — The normality of the data was tested employing Shapiro test, and the differences between means was done using the T-test for normal data and the Wilcox test for non-normal data. [file elife-64581-code1.pdf.zip › STATISTICAL_ANALYSIS_GLYPICANSHh_RECRUITMENT.pdf]

STATISTICAL ANALYSIS OF DALLY RECRUITMENT

```
R version 3.5.2 (2018-12-20) -- "Eggshell Igloo"
Copyright (C) 2018 The R Foundation for Statistical Computing
Platform: i386-w64-mingw32/i386 (32-bit)

R is free software and comes with ABSOLUTELY NO WARRANTY.
You are welcome to redistribute it under certain conditions.
Type 'license()' or 'licence()' for distribution details.

R is a collaborative project with many contributors.
Type 'contributors()' for more information and
'citation()' on how to cite R or R packages in publications.

Type 'demo()' for some demos, 'help()' for on-line help, or
'help.start()' for an HTML browser interface to help.
Type 'q()' to quit R.

> library(readxl)
> DallyR <- read_excel("C:/Users/USUARIO/Desktop/FOTOS NUEVAS/CUANTIFI
CACIÓN GLIPICANOS/DallyR.xlsx")
> View(DallyR)
> attach(DallyR)
> Trat=split(DallyR$Dll,DallyR$Tratamiento,drop=FALSE)
> shapiro.test(Trat$Ihog)

        Shapiro-Wilk normality test

data:  Trat$Ihog
W = 0.91208, p-value = 0.4105

> shapiro.test(Trat$DFN)

        Shapiro-Wilk normality test

data:  Trat$DFN
W = 0.96125, p-value = 0.8294

> shapiro.test(Trat$DFN1)

        Shapiro-Wilk normality test

data:  Trat$DFN1
W = 0.97862, p-value = 0.9526

> shapiro.test(Trat$DFN2)

        Shapiro-Wilk normality test

data:  Trat$DFN2
W = 0.98548, p-value = 0.9818

> shapiro.test(Trat$DIg)

        Shapiro-Wilk normality test

data:  Trat$DIg
W = 0.92607, p-value = 0.518
```

```
pairwise.t.test(Dll,Tratamiento, paired = FALSE)

        Pairwise comparisons using t tests with pooled SD

data:  Dll and Tratamiento

       DFN     DFN1    DFN2    DIg   FN1+++
DFN1   0.027   -       -       -     -
DFN2   0.023   1.000   -       -     -
DIg    4.7e-10 6.1e-07 8.1e-07 -     -
FN1+++ 1.0e-09 1.0e-06 1.3e-06 1.000 -
Ihog   9.9e-11 1.1e-07 1.5e-07 1.000 1.000

P value adjustment method: holm
```

STATISTICAL ANALYSIS OF DLP RECRUITMENT

```
R version 3.5.2 (2018-12-20) -- "Eggshell Igloo"
Copyright (C) 2018 The R Foundation for Statistical Computing
Platform: i386-w64-mingw32/i386 (32-bit)

R is free software and comes with ABSOLUTELY NO WARRANTY.
You are welcome to redistribute it under certain conditions.
Type 'license()' or 'licence()' for distribution details.

R is a collaborative project with many contributors.
Type 'contributors()' for more information and
'citation()' on how to cite R or R packages in publications.

Type 'demo()' for some demos, 'help()' for on-line help, or
'help.start()' for an HTML browser interface to help.
Type 'q()' to quit R.

> library(readxl)
> DallylikeR <- read_excel("C:/Users/USUARIO/Desktop/FOTOS NUEVAS/CUAN
TIFICACIÓN GLIPICANOS/DallylikeR.xlsx")
> View(DallylikeR)
> attach(DallylikeR)
> Trat=split(DallylikeR$DLP,DallylikeR$Tratamiento,drop=FALSE)
> shapiro.test(Trat$Ihog)

        Shapiro-Wilk normality test

data:  Trat$Ihog
W = 0.86281, p-value = 0.1604

> shapiro.test(Trat$DFN)

        Shapiro-Wilk normality test

data:  Trat$DFN
W = 0.87104, p-value = 0.1896

> shapiro.test(Trat$DFN1)
```

```
        Shapiro-Wilk normality test

data:  Trat$DFN1
W = 0.90397, p-value = 0.3557

> shapiro.test(Trat$DFN2)

        Shapiro-Wilk normality test

data:  Trat$DFN2
W = 0.91788, p-value = 0.4531

> shapiro.test(Trat$`FN1+++`)

        Shapiro-Wilk normality test

data:  Trat$`FN1+++`
W = 0.97769, p-value = 0.9219
> shapiro.test(Trat$Dig)

        Shapiro-Wilk normality test

data:  Trat$Dig
W = 0.90305, p-value = 0.3498


> pairwise.t.test(DLP,Tratamiento)

        Pairwise comparisons using t tests with pooled SD

data:  DLP and Tratamiento

       DFN     DFN1   DFN2   Dig    FN1+++
DFN1   0.0324  -      -      -      -
DFN2   6.4e-06 0.0288 -      -      -
Dig    6.4e-07 0.0037 1.0000 -      -
FN1+++ 1.3e-06 0.0037 1.0000 1.0000 -
Ihog   1.8e-06 0.0094 1.0000 1.0000 1.0000

P value adjustment method: holm
```

STATISTICAL ANALYSIS OF HH RECRUITMENT

```
R version 3.5.2 (2018-12-20) -- "Eggshell Igloo"
Copyright (C) 2018 The R Foundation for Statistical Computing
Platform: i386-w64-mingw32/i386 (32-bit)

R is free software and comes with ABSOLUTELY NO WARRANTY.
You are welcome to redistribute it under certain conditions.
Type 'license()' or 'licence()' for distribution details.

R is a collaborative project with many contributors.
Type 'contributors()' for more information and
'citation()' on how to cite R or R packages in publications.

Type 'demo()' for some demos, 'help()' for on-line help, or
'help.start()' for an HTML browser interface to help.
Type 'q()' to quit R.
```

```
> library(readxl)
> HhR <- read_excel("C:/Users/USUARIO/Desktop/FOTOS NUEVAS/CUANTIFICAC
IÓN HH/HhR.xlsx")
> View(HhR)
> attach(HhR)
> Trat=split(HhR$Hh,HhR$Tratamiento,drop=FALSE)
> shapiro.test(Trat$Ihog)

        Shapiro-Wilk normality test

data:  Trat$Ihog
W = 0.91411, p-value = 0.464

> shapiro.test(Trat$DFN)

        Shapiro-Wilk normality test

data:  Trat$DFN
W = 0.94515, p-value = 0.6855

> shapiro.test(Trat$DFN1)

        Shapiro-Wilk normality test

data:  Trat$DFN1
W = 0.83649, p-value = 0.09214

> shapiro.test(Trat$DFN2)

        Shapiro-Wilk normality test

data:  Trat$DFN2
W = 0.94824, p-value = 0.726

> shapiro.test(Trat$`FN1 Cherry`)

        Shapiro-Wilk normality test

data:  Trat$`FN1 Cherry`
W = 0.87792, p-value = 0.2174

> shapiro.test(Trat$`FN1+++`)

        Shapiro-Wilk normality test

data:  Trat$`FN1+++`
W = 0.97907, p-value = 0.9549

> pairwise.t.test(Hh,Tratamiento)

        Pairwise comparisons using t tests with pooled SD

data:  Hh and Tratamiento

           DFN     DFN1    DFN2    FN1 Cherry FN1+++
DFN1       1.000   -       -       -          -
DFN2       3.3e-06 9.6e-06 -       -          -
FN1 Cherry 0.012   0.027   0.027   -          -
FN1+++     1.000   1.000   9.6e-06 0.027      -
Ihog       < 2e-16 < 2e-16 < 2e-16 < 2e-16    < 2e-16

P value adjustment method: holm
```

```
> pairwise.t.test(Hh,Tratamiento, paired = FALSE)

        Pairwise comparisons using t tests with pooled SD

data:  Hh and Tratamiento

           DFN     DFN1    DFN2    FN1 Cherry FN1+++
DFN1       1.000   -       -       -          -
DFN2       3.3e-06 9.6e-06 -       -          -
FN1 Cherry 0.012   0.027   0.027   -          -
FN1+++     1.000   1.000   9.6e-06 0.027      -
Ihog       < 2e-16 < 2e-16 < 2e-16 < 2e-16    < 2e-16

P value adjustment method: holm
```
