# Supplementary material for: Glypicans define unique roles for the Hedgehog co-receptors boi and ihog in cytoneme-mediated gradient formation
Source: eLife. 2021 Aug 6;10:e64581. doi: 10.7554/eLife.64581 (PMC8410076; doi:10.7554/eLife.64581)
Supplement: Source code 2. — For genotypes showing only dynamic cytonemes, the statistical analysis was done using their numerical lifetimes. To examine the normality of the data distribution, we performed a Shapiro–Wilk test. Since the results showed a non-parametric distribution of the experimental data, we selected a Wilcoxon rank sum test to compare the numerical lifetimes between two genotypes. For genotypes showing both static and non-static cytonemes we defined a no numerical case to quantify the frequency of static cytonemes. As a result, we obtained ‘mixed’ violin plots representing the distribution of the lifetime of the whole cytoneme population. [file elife-64581-code2.pdf.zip › STATISTICAL_ANALYSIS_CYTONEMES_DYNAMICS.pdf]

```
% Simulation parameters

% To import the data upload the excel file to the command window of matlab

% Using import window of matlab, select the data as numeric matrix with the following
conditions (in that specidfic order):

%  Replace -- Blanks with -- 10000
%  Replace -- unimportable cells with -- NaN

data=MutantsIhog; % Name of the loaded data file.

cases=8; % Number of genotypes to study.

TM=30; % Time of movies (in min).
% This value is the one consider as static in the final graph for data > TM

bw=1; % band width = precicion of the violin plots.


%% Script
format shortE

d=NaN(1,cases);
for i=1:cases
    %Size per phenotype
    temp=find(data(:,i)==10000,1);

    if isempty(temp)==1
        d(i)=size(data(:,i),1);
    else
        d(i)=temp-1;
    end

end

% Definition of lifetimes group per genotype
Times=cell(1,cases);
for i=1:cases
   Times{i}=data(1:d(i),i);
end


% To consider static cases for representation purposes the times bigger than TM (NaN
values) are fixed to TM.
Ttot=Times;
for j=1:cases
    for l=1:d(j)
        if isnan(Times{j}(l))==1
            Ttot{j}(l)=TM;
        end
    end
end
```

```
%% Statistical analysis

% Study of normality of the data.

% Shapiro-Wilk test is not implemented in Matlab code, for the statistical study of↙
normality we used the scrip developed by:

% Copyright (c) 17 March 2009 by Ahmed Ben Saïda,
% Department of Finance, IHEC Sousse - Tunisia
% https://es.mathworks.com/matlabcentral/fileexchange/13964-shapiro-wilk-and-shapiro-↙
francia-normality-tests

SW=NaN(1,cases);

for n=1:cases
    SW(n)=swtest(Times{n},0.05);
end

% Depend on the results of SW test a Ttest or Wilcoxon test is used for
% the satitistical analysis:
PValues=NaN(cases);

if sum(SW)==0
    for i=1:cases
        for j=i+1:cases
           [temp,temp2]=ttest2(Times{i},Times{j});
           PValues(i,j)=temp2;
        end
    end

else

    for i=1:cases
       for j=i+1:cases
          PValues(i,j)=ranksum(Times{i},Times{j});
       end
    end

end

if sum(SW)==0
    disp('P values of statistical study (T-test) for lifetimes: ');
else
    disp('P values of statistical study (Wilcoxon test) for lifetimes: ');
end

disp(PValues);


%% Graphical script


% Violin plot function is not implemented in Matlab code, for the the representation↙
of the data we used the scrip developed by:
```

```
% Hoffmann H, 2015: violin.m - Simple violin plot using matlab default kernel density↙
estimation. INRES (University of Bonn), Katzenburgweg 5, 53115 Germany.
% https://es.mathworks.com/matlabcentral/fileexchange/45134-violin-plot


figure % Plots Violin of the data
    Violin(Times,'bw',bw);
    ylabel('Time (min)','Fontsize',18)
    title('Violins of cytoneme lifetimes')

figure % Plots a Violin considering the statical phenotype
    Violin(Ttot,'bw',bw);
    ylabel('Time (min)','Fontsize',18)
    title('Violins of cytoneme lifetimes (including static cytonemes)')
```
