# Supplementary material for: Glypicans define unique roles for the Hedgehog co-receptors boi and ihog in cytoneme-mediated gradient formation
Source: eLife. 2021 Aug 6;10:e64581. doi: 10.7554/eLife.64581 (PMC8410076; doi:10.7554/eLife.64581)
Supplement: Transparent reporting form [file elife-64581-transrepform.docx]

***eLife’s* transparent reporting form**

We encourage authors to provide detailed information *within their submission* to facilitate the interpretation and replication of experiments. Authors can upload supporting documentation to indicate the use of appropriate reporting guidelines for health-related research (see [EQUATOR Network](http://www.equator-network.org/%20)), life science research (see the [BioSharing Information Resource](https://biosharing.org/)), or the [ARRIVE guidelines](http://www.plosbiology.org/article/info:doi/10.1371/journal.pbio.1000412) for reporting work involving animal research. Where applicable, authors should refer to any relevant reporting standards documents in this form.

If you have any questions, please consult our Journal Policies and/or contact us: [editorial@elifesciences.org](mailto:editorial@elifesciences.org).

**Sample-size estimation**

- You should state whether an appropriate sample size was computed when the study was being designed
- You should state the statistical method of sample size computation and any required assumptions
- If no explicit power analysis was used, you should describe how you decided what sample (replicate) size (number) to use

Please outline where this information can be found within the submission (e.g., sections or figure legends), or explain why this information doesn’t apply to your submission:

Sample size depends on experimental subjects available. Experiments were setup to provide a minimum sample size according to our previous published research. The sample size is available in the figure legends and the number of samples is detailed in “Material and methods”> “Quantification method and numerical analysis of cytoneme dynamics” for cytoneme dynamics, and in “Material and methods”> “Quantification method and numerical analysis of glypicans and Hh recruitment” for Hh, Dally and Dlp measures.

**Replicates**

- You should report how often each experiment was performed
- You should include a definition of biological versus technical replication
- The data obtained should be provided and sufficient information should be provided to indicate the number of independent biological and/or technical replicates
- If you encountered any outliers, you should describe how these were handled
- Criteria for exclusion/inclusion of data should be clearly stated
- High-throughput sequence data should be uploaded before submission, with a private link for reviewers provided (these are available from both GEO and ArrayExpress)

Please outline where this information can be found within the submission (e.g., sections or figure legends), or explain why this information doesn’t apply to your submission:

The number of replicates is available in figure legends, “Material and methods”, and in “Source data” excels. The criterium to include data was that the same result was obtained with different replicates of the experiment.

**Statistical reporting**

- Statistical analysis methods should be described and justified
- Raw data should be presented in figures whenever informative to do so (typically when N per group is less than 10)
- For each experiment, you should identify the statistical tests used, exact values of N, definitions of center, methods of multiple test correction, and dispersion and precision measures (e.g., mean, median, SD, SEM, confidence intervals; and, for the major substantive results, a measure of effect size (e.g., Pearson's r, Cohen's d)
- Report exact p-values wherever possible alongside the summary statistics and 95% confidence intervals. These should be reported for all key questions and not only when the p-value is less than 0.05.

Please outline where this information can be found within the submission (e.g., sections or figure legends), or explain why this information doesn’t apply to your submission:

In the case of cytoneme dynamics, to examine the normality of the data distribution we performed a Shapiro-Wilk test. Since the results showed a non-parametric distribution of the experimental data, we selected a Wilcoxon rank sum test to compare the numerical lifetimes between two genotypes.

This information is detailed explained in “Material and methods”> “Quantification method and numerical analysis of cytoneme dynamics”. We have also attached an Excel file with the raw data and PDF with a detailed Matlab script.

In the case of Hh, Dally and Dlp measures, to examine the normality of the data distribution we performed a Shapiro-Wilk test. Since the results showed both parametric and non-parametric distribution of the experimental data, we selected a Wilcoxon rank sum test to compare the numerical lifetimes between two genotypes of non-parametric data and a T-test for parametric data.

This information is detailed explained in “Material and methods”> “Quantification method and numerical analysis of glypicans and Hh recruitment”. We have also attached an Excel file with the raw data and PDF with a detailed R-studio analysis.

(For large datasets, or papers with a very large number of statistical tests, you may upload a single table file with tests, Ns, etc., with reference to sections in the manuscript.)

**Group allocation**

- Indicate how samples were allocated into experimental groups (in the case of clinical studies, please specify allocation to treatment method); if randomization was used, please also state if restricted randomization was applied
- Indicate if masking was used during group allocation, data collection and/or data analysis

Please outline where this information can be found within the submission (e.g., sections or figure legends), or explain why this information doesn’t apply to your submission:

The statistical analysis was performed over groups organized according to genotypes. The full data of cytoneme dynamics was statistical compared between them using a Wilcoxon rank sum test from Matlab.

The full data of Hh, Dally and Dlp measures was statistical compared between them using a T-test or a Wilcoxon test from R-studio.

This information is detailed explained in “Material and methods”> “Quantification method and numerical analysis of cytoneme dynamics” and “Material and methods”> “Quantification method and numerical analysis of glypicans and Hh recruitment”.

The full raw data are submitted in a source data file.

**Additional data files (“source data”)**

- We encourage you to upload relevant additional data files, such as numerical data that are represented as a graph in a figure, or as a summary table
- Where provided, these should be in the most useful format, and they can be uploaded as “Source data” files linked to a main figure or table
- Include model definition files including the full list of parameters used
- Include code used for data analysis (e.g., R, MatLab)
- Avoid stating that data files are “available upon request”

Please indicate the figures or tables for which source data files have been provided:
